# Comparison of overall survival in patients with unresectable hepatic metastases with or without transarterial chemoembolization: A Propensity Score Matching Study

**DOI:** 10.1038/srep35336

**Published:** 2016-10-13

**Authors:** F. Y. Wang, W. Meng, Y. Li, T. Li, C. Y. Qin

**Affiliations:** 1 Department of gastroenterology and hepatology, Shandong Provincial Hospital, Shandong University, Shandong, 250100, China; 2 Department of ECG, Qilu hospital affiliated to Shandong University, Shandong, 250012, China; 3 Department of Intensive care unit, Qilu hospital affiliated to Shandong University, Shandong, 250012, China; 4 Department of Gastroenterology and Hepatology, Shandong Province Hospital Affiliated to Shandong University, Shandong, 250021, China; 5 Shandong provincial engineering and technological research center for liver diseases prevention and control, Shandong, 250021, China.

## Abstract

Transarterial chemoembolization (TACE) has mostly been used in hypervascular tumours such as hepatocellular carcinoma, and may be an effective palliative treatment in patients with metastatic liver cancer. Our goal is to determine whether TACE increases overall survival (OS) of in patients with liver metastases. The retrospective cohort study included 171 patients with liver metastases diagnosed between 2001 and 2015. OS was compared between the TACE and non-TACE groups after propensity score matching to reduce the effects of selection bias and potential confounders. Multivariate analysis was conducted to confirm the confounding factors with OS. After excluding 43 patients, 128 patients were analysed and among thses 64 patients (50%) were included in the TACE group. In the propensity score matched cohort (42 pairs), the OS was non-significantly longer in the TACE group than in the non-TACE group (*p* = 0.789). Multivariate analysis revealed that international normalized ratio (INR) (HR 0.058, 95%CI: [0.005, 0.681]; *p* = 0.023) and Radiofrequency ablation (RFA) (HR 3.054, 95%CI: [1.418, 6.579]; *p* = 0.004) were independent risk factors for OS in patients with unresectable liver metastases. There were no significant differences in patients with unresectable liver metastases with or without TACE. INR and RFA can significantly affect OS in patients with unresectable liver metastases.

Metastatic liver disease represents a common challenge in oncology. The liver is the most common site of metastases that arise from gastrointestinal malignancies^1^; and, other primary sites of origin, including breast, lung, pancreas, and endometrial carcinomas. Local therapy for liver metastases from primary locations, such as breast, lung, gastric or pancreatic cancer may have little success due to the presence of extrahepatic disease. For colorectal cancer, hepatic resection in selected patients can result in 5-year survival rates of 20% to 44–45%^2^,^3^. However, compared with the number of patients demonstrating liver metastasis, the number of resectable candidates is limited.

In most cases, liver metastases are treated with oral or intravenous chemotherapy. Transarterial chemoembolization (TACE), the combination of the injection of a drug and embolic material, has mostly been used in hypervascular tumours such as hepatocellular carcinoma^4^, and may be an effective palliative treatment in patients with metastatic liver cancer.

Propensity score matching^5^ is a statistical technique in which a treatment case is matched with one or more control cases based on each case’s propensity score. This matching can help strengthen causal arguments in quasi-experimental and observational studies by reducing selection bias.

The present retrospective study aimed to evaluate overall survival (OS) outcome in patients with or without TACE.

## Results

### Patient Characteristics before Propensity Score Matching

A total of 128 patients with unresectable hepatic metastases were included in the study; 64 patients (50%) were included in the TACE group and the remaining 64 were included in the non-TACE group.

The baseline characteristics of the TACE and non-TACE groups are summarized in Table 1. There were no significant differences between the two groups with respect to age, gender, primary tumour sites, other transfers, hypertension, diabetes, CHD, smoking, AST, PT, TBIL, albumin, Child-Pugh score, Child-Pugh classification, INR, ascites, and RFA. However, the numbers of hepatic metastases (p = 0.044), hepatitis (p = 0.043) and APTT (p = 0.003) were significantly different between the TACE and non-TACE groups.

### Patient Characteristics after Propensity Score Matching

In the propensity score matched cohort, there were no significant differences between the two groups regarding age, gender, primary tumour site, numbers of hepatic metastases, other transfers, hepatitis, hypertension, diabetes, CHD, smoking, AST, PT, APTT, TBIL, albumin, Child-Pugh score, Child-Pugh classification, INR, ascites, and RFA. The results were showed in Table 2.

### OS After Propensity Score Matching

**Figure 1:**
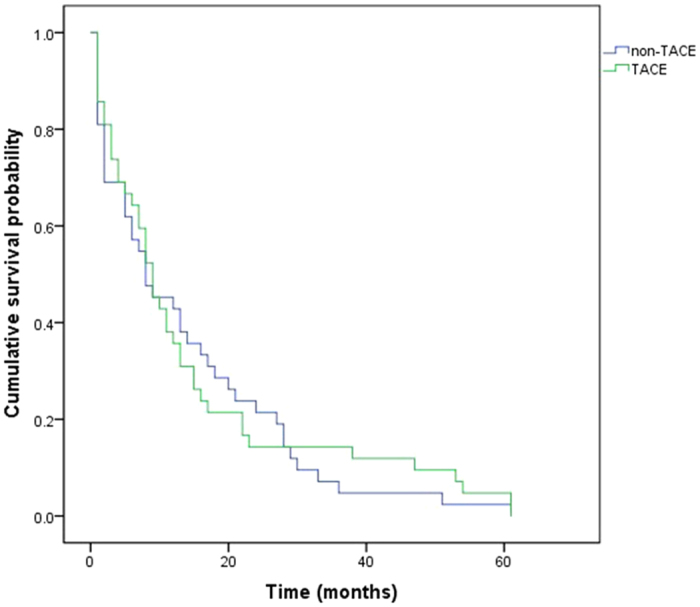
Kaplan-Meier analysis of OS between the two groups.

Kaplan-Meier survival analysis indicated no significant difference in median OS between the TACE and non-TACE groups (9, 95%CI: [6.29, 11.71]) vs. (8, 95%CI: [1.656, 14.344]) months, respectively; *p* = 0.789). Thus, TACE did not have a significant influence on OS. The result was showed in Fig. 1.

### Multivariate analysis for the association of confounding factors with OS

To adjust for the simultaneous impact of potential confounders, Cox proportional hazards regression was performed (Table 3). In the univariate analysis, age, Child-Pugh score, Child-Pugh classification, AST, TBIL, albumin, APTT, INR and RFA were associated with OS. Multivariate analysis revealed that INR (HR 0.058, 95%CI: [0.005, 0.681]; *p* = 0.023) and RFA (HR 3.054, 95%CI: [1.418, 6.579]; *p* = 0.004) were independent risk factors for OS.

## Discussion

The liver is the most common site metastasis from tumours that initially arise in colorectal cancer^6^. Twenty-five percent of the patients were diagnosed with liver metastases when they were found colorectal cancer. Surgery can improve the 5-year survival for resectable liver-only metastases of colorectal cancer^7^,^8^. In a meta-analysis of observational studies, Luca Martella *et al*.^9^ found surgery showed a survival advantage for hepatic metastases of gastric cancer. However, many patients lose their chance for surgery when liver metastases are found. Our study researched patients with unresectable liver metastases; however, our patients’ primary cancers were not limited to gastric and colorectal cancer.

In a study by Albert M. *et al*.^10^, TACE (with cisplatin, doxorubicin, mitomycin C, ethiodol and polyvinyl alcohol) for colorectal liver metastases provided local disease control of hepatic metastases after 43% of treatment cycles, with a median survival of 27 months overall. Their study included patients with unresectable liver metastases or recurrence after surgical resection. Hong K *et al*.^11^ found that median survival times was 7.7 months for TACE. In our research, the primary cancer included pancreas, stomach, endometrium, colorectum, ovaries, bile duct, lung, kidney, duodenum, breast, oesophagus, jejunum, gallbladder, and mouth. Furthermore, we excluded patients who previously had local liver surgery. Patients were further excluded if the primary cancer was leukemia, lymphoma or melanoma.

We found that median OS was 9 months and 8 months in the TACE and non-TACE groups, respectively, and that there were no significant differences in either group (*p* = 0.789).

Gunduz S *et al*.^12^ found that INR values reflecting the functional hepatic reserve can be used as a positive predictive factors for median hepatic progression-free survival with unresectable liver metastases. We found that INR could have a significant influence on the OS of unresectable liver metastases (*p* = 0.005).

Local ablative therapy for the treatment of metastatic liver disease has been evaluated most extensively in colorectal cancer with 5-year survival rates up to 55% after RFA^6^. Nielsen K *et al*.^13^ proved that RFA of colorectal liver metastases, after conversion chemotherapy, provides potential local control and good OS. Jakobs TF *et al*.^14^ proposed that RFA might improve survival for patients with unresectable hepatic metastases of colorectal cancer. In our research, we also proved that RFA was an effective means to alleviate unresectable liver metastases.

There are limitations to the present study due to its retrospective design. There were 22 patients who were not included in the matched cohort analysis in the TACE group. More patients for the non-TACE group were needed to match more pairs. Cancer-free survival, local recurrence, and adverse events should be investigated in the future.

In conclusion, our propensity matching score study suggests no significant difference in unresectable liver metastases with or without TACE. Further, INR and RFA can significantly affect OS of patients with unresectable liver metastases.

## Methods

### Patients

This retrospective cohort study included 171 hepatic metastases patients at Qilu hospital affiliated with Shandong University, Shandong, China and Shandong Provincial Hospital, Shandong, China from 2001 to 2015. The primary cancer sites of hepatic metastases included pancreas, stomach, endometrium, colorectum, ovaries, bile duct, lung, kidney, duodenum, breast, oesophagus, jejunum, gallbladder, and mouth. Patients who met any of the following criteria were excluded: (i) the primary cancer weas melanoma or a haemal tumour, (ii) liver cancer was the origin cancer, (iii) patients who underwent a liver resection, (iv) patients who underwent TACE therapy in other hospitals, (v) patients who refused further therapy after they were diagnosed with liver metastases, and (vi) patients who did not participate in the follow-up process. Based on these criteria, a total of 43 patients were excluded from the study. Of these, the primary cancer of 11 patients was melanoma or haemal tumour, 15 patients had undergone liver resections, 2 patients refused further therapy, 4 patients underwent TACE in other hospitals, 3 patients had liver cancer as the primary cancer, and 8 patients were did not participate in follow-up process. Finally, a total of 128 patients were included in our study.

**Figure 2:**
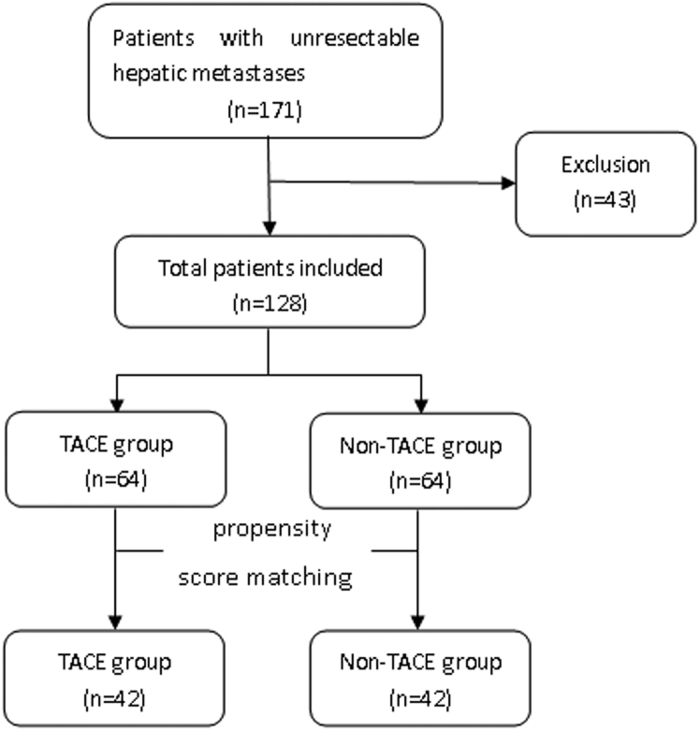
Flowchart of the study inclusion protocol.

To reduce the effects of selection bias and potential confounders in this study, we performed rigorous adjustment for differences in baseline characteristics by using propensity score matching. We considered age, gender, primary tumour sites, numbers of hepatic metastases, other transfers, hypertension, diabetes, coronary heart disease (CHD), smoking, hepatitis, Aspartate transaminase (AST), prothrombin time (PT), total bilirubin(TBIL), albumin, Child-Pugh score, Child-Pugh classification, activated partial thromboplastin time (APTT), international normalized ratio(INR), ascites, and radiofrequency ablation (RFA). 42 patients’ pairs were selected (Fig. 2). The study protocols were conducted in accordance with the Declaration of Helsinki and current ethical guidelines. Our study was approved by the Medical Ethics Committee of Shandong Provincial hospital and informed consent was obtained from all subjects.

### Data collection and follow up

The following demographic, laboratory and clinical information was collected from medical chart review: age, gender, primary tumour sites, numbers of hepatic metastases, other transfers, hypertension, diabetes, CHD, smoking, hepatitis, AST, PT, TBIL, albumin, Child-Pugh score, Child-Pugh classification, APTT, INR, ascites, and RFA. Survival outcome and other patient information was obtained mostly by telephone follow-up. The survival time was defined from diagnosis of liver metastases to death or loss of follow-up.

### Propensity Score Analysis

The propensity scores were estimated with all variables presented in Table 1 (baseline characteristics) using a parsimonious logistic regression model. We used the nearest neighbor matching algorithm without replacement. One to one^15^ calliper matching was performed within 25% of the standard deviation of the log-trans-formed propensity scores. The value of caliper was 0.5. In the propensity score-matched cohort, the two groups were compared in terms of baseline characteristics. The balance of the matched cohort was evaluated using standardized mean difference and hypothetical test. The Kaplan-Meier method with a log-rank test was applied to compare the survival distributions of patients. Cox proportional hazards regression was used to examine the association of TACE with survival rates by adjusting for the simultaneous impact of potential confounders. Multivariate analysis was performed on variables that were associated with survival rates based on univariate analysis (P < 0.05). Hazard ratios with 95% confidence intervals (CIs) were calculated.

### Statistical Methods

In all study subjects, continuous variables were compared parametrically using Student’s *t*-test or non-parametrically using the Mann-Whitney *U*-test. Categorical variables were compared using the *χ*^*2*^-test or Fisher’s exact test as appropriate.

Statistical results are presented as the mean ± s.d., and number of patients(%). Two-sided tests, *P* values < 0.05 were taken as significant. Statistical analyses were conducted using the IBM SPSS statistical package 22.0 (IBM, Armonk, NY, USA) with three plug-in (SPSS R-plug-in, R and psmatching).

## Additional Information

**How to cite this article**: Fengyan, W. *et al*. Comparison of overall survival in patients with unresectable hepatic metastases with or without transarterial chemoembolization: A Propensity Score Matching Study. *Sci. Rep.* **6**, 35336; doi: 10.1038/srep35336 (2016).

## Figures and Tables

**Table 1:** Comparison of baseline characteristics between the TACE (n = 64) and non-TACE (n = 64) groups before propensity score matching.

Variables	TACE group	non-TACE group	*p* value
Age (years)	60.44 ± 10.65	59.34 ± 11.92	0.585
Gender, male	41 (64.1%)	41 (64.1%)	1
Primary tumour site			
Pancreas	7 (10.9%)	8 (12.5%)	0.688
Stomach	17 (26.6%)	17 (26.6%)	
Endometrium	1 (1.6%)	0	
Chorion	1 (1.6%)	0	
Colorectum	19 (29.7%)	17 (26.6%)	
Ovaries	2 (3.1%)	2 (3.1%)	
Bile ducts	4 (6.3%)	0	
Lung	4 (6.3%)	6 (9.4%)	
Kidney	0	2 (3.1%)	
Duodenum	3 (4.7%)	2 (3.1%)	
Breast	1 (1.6%)	2 (3.1%)	
Oesophagus	1 (1.6%)	3 (4.7%)	
Gallbladder	0	1 (1.6%)	
Others	4 (6.3%)	4 (6.3%)	
Numbers of hepatic metastases			
One	4 (6.3%)	10 (15.6%)	0.044
Two	2 (3.1%)	3 (4.7%)	
More	43 (67.2%)	46 (71.9%)	
Unknown	15 (23.4%)	5 (7.8%)	
Other transfers			
Lymph gland	9 (14.1%)	16 (25%)	0.145
Other organs	12 (18.8%)	16 (25%)	
Unknown	43 (67.2%)	32 (50%)	
Hypertension	14 (21.9%)	14 (21.9%)	1
Diabetes	7 (10.9%)	4 (6.3%)	0.344
CHD	7 (10.9%)	5 (7.8%)	0.544
Smoking	20 (31.3%)	26 (40.6.90%)	0.269
Hepatitis	1 (1.6%)	0	0.043
Child-Pugh score	5.25 ± 0.69	5.13 ± 0.49	0.239
Child-Pugh classification			
A	59 (92.2%)	62 (96.9%)	
B	5 (7.8%)	2 (3.1%)	
AST	33.80 ± 19.52	37.09 ± 33.50	0.498
TBIL	16.53 ± 16.47	18.95 ± 32.47	0.596
Albumin	39.87 ± 5.56	38.71 ± 6.26	0.271
PT	13.51 ± 14.61	12.09 ± 1.74	0.44
APTT	40.05 ± 24.27	30.56 ± 4.99	0.003
INR	1.02 ± 0.11	1.03 ± 0.11	0.522
Ascites	2 (3.1%)	3 (4.7%)	0.648
RFA	8 (12.5%)	9 (14.1%)	0.795

Abbreviations: CHD, coronary heart disease; AST, aspartate aminotransferase; APTT, activated partial thromboplastin time; INR, international normalized ratio; RFA, Radiofrequency ablation. Data are shown as the mean ± s.d. or number (%) of patients.

**Table 2:** Comparison of baseline characteristic between the TACE (n = 42) and non-TACE (n = 42) groups after propensity score matching.

Variables	TACE group	non-TACE group	*p* value
Age (years)	60.74 ± 9.57	58.38 ± 11.22	0.303
Gender, male	30 (71.4%)	26 (61.9%)	0.355
Primary tumour site			
Pancreas	6 (14.3%)	5 (11.9%)	0.977
Stomach	12 (28.6%)	11 (26.2%)	
Endometrium	0	0	
Chorion	0	0	
Colorectum	11 (26.2%)	11 (26.2%)	
Ovaries	1 (2.4%)	2 (4.8%)	
Bile ducts	2 (4.8%)	0	
Lung	3 (7.1%)	2 (4.8%)	
Kidney	0	1 (2.4%)	
Duodenum	2 (4.8%)	2 (4.8%)	
Breast	1 (2.4%)	2 (4.8%)	
Oesophagus	1 (2.4%)	2 (4.8%)	
Gallbladder	0	1 (2.4%)	
Others	3 (7.1%)	3 (7.1%)	
Numbers of hepatic metastases			
One	2 (4.8%)	6 (14.3%)	0.085
Two	0	2 (4.8%)	
More	30 (71.4%)	30 (71.4%)	
Unknown	10 (23.8%)	4 (9.5%)	
Other transfers			
Lymph gland	6 (14.3%)	11 (26.2%)	0.088
Other organs	6 (14.3%)	11 (26.2%)	
Unknown	30 (71.4%)	20 (47.6%)	
Hypertension	9 (21.4%)	10 (23.8%)	0.794
Diabetes	3 (7.1%)	2 (4.8%)	0.645
CHD	3 (7.1%)	3 (7.1%)	1
Smoking	16 (38.1%)	17 (40.5%)	0.823
Hepatitis	0	0	
Child-Pugh score	5.24 ± 0.69	5.14 ± 0.57	0.492
Child-Pugh classification			
A	38 (90.5%)	40 (95.2%)	0.676
B	4 (9.5%)	2 (4.8%)	
AST	32.14 ± 19.25	39.19 ± 38.80	0.295
TBIL	16.49 ± 17.66	22.53 ± 39.63	0.369
Albumin	39.77 ± 5.59	37.88 ± 6.35	0.152
PT	14.20 ± 18.04	12.11 ± 1.79	0.456
APTT	30.12 ± 3.13	29.69 ± 3.20	0.536
INR	1.01 ± 0.10	1.04 ± 0.12	0.184
Ascites	0	1 (2.4%)	0.314
RFA	5 (11.9%)	5 (11.9%)	1

Abbreviations: CHD, coronary heart disease; AST, aspartate aminotransferase; APTT, activated partial thromboplastin time; INR, international normalized ratio; RFA, Radiofrequency ablation. Data are shown as the mean ± s.d. or number (%) of patients.

**Table 3:** Cox proportional hazards multivariate regression analysis of OS.

Viarables	Hazard Ratio (95% CI)	*p* value
Age	1.007 (0.982, 1.033)	0.585
C-P score	2.050 (0.575, 7.307)	0.268
C-P classification	3.319 (0.242, 45.535)	0.369
AST	1.010 (0.999, 1.021)	0.075
TBIL	1.001 (0.990, 1.013)	0.829
Albumin	0.968 (0.920, 1.019)	0.217
APTT	1.014 (0.935, 1.099)	0.739
INR	0.058 (0.005, 0.681)	0.023
RFA	3.054 (1.418, 6.579)	0.004
TACE	1.075 (0.625, 1.849)	0.795

Cox proportional hazards multivariate regression analysis of overall survival.
